# Complete Remission After Percutaneous Renal Artery Angioplasty for Focal Segmental Glomerulosclerosis due to Takayasu Disease: A Case Report

**DOI:** 10.1016/j.xkme.2026.101251

**Published:** 2026-01-08

**Authors:** Yusuke Ushio, Shun Manabe, Anna Nakai, Momoko Seki, Shiho Makabe, Shizuka Kobayashi, Yuki Kawaguchi, Hiroshi Kataoka, Naoko Ito, Sekiko Taneda, Kazuho Honda, Junichi Hoshino

**Affiliations:** 1 Department of Nephrology, Tokyo Women’s Medical University, Tokyo, Japan; 2 Department of Surgical Pathology, Tokyo Women’s Medical University, Tokyo, Japan; 3 Department of Diagnostic Pathology, Showa Medical University School of Medicine, Tokyo, Japan

**Keywords:** Takayasu disease, percutaneous transluminal renal angioplasty, focal segmental glomerulosclerosis, renal artery stenosis

## Abstract

Renal artery stenosis is a common complication of Takayasu disease; however, the presence of nephrotic syndrome is rare. Percutaneous transluminal renal angioplasty (PTRA) is performed for renal artery stenosis; however, its efficacy is unclear for renal artery stenosis complicated with nephrotic syndrome. This is a case report of Takayasu disease in an 18-year-old woman with no relevant medical history. Magnetic resonance angiography revealed bilateral renal artery stenosis, and nephrotic syndrome was noted. Focal segmental glomerulosclerosis was diagnosed based on renal biopsy. As atrophy was observed in the right kidney, PTRA was performed for the left renal artery stenosis. Urine protein levels decreased subsequently, leading to remission. Renal artery stenosis may commonly occur due to Takayasu disease; however, the resulting accentuation of the renin-angiotensin-aldosterone cascade and increased intraglomerular pressure may cause focal segmental glomerulosclerosis. PTRA may induce remission if renal dysfunction is mild, and it should be performed as early as possible.

Renal artery stenosis is a common symptom of Takayasu disease.^1^ The development of nephrotic syndrome with renal artery stenosis is frequently observed.^2^, ^3^, ^4^, ^5^ However, it rarely presents with Takayasu disease, and limited cases have been reported.^6^ Percutaneous transluminal renal angioplasty (PTRA) is normally performed for renal artery stenosis accompanied by renovascular hypertension; however, only a few reports have noted its effectiveness for cases with nephrotic syndrome.^7^^,^^8^

Herein, we describe a case of renal artery stenosis due to Takayasu disease in which complete remission of nephrotic syndrome was achieved following PTRA.

### Case Report

An 18-year-old woman presenting with a 4-month history of pyrexia and arthralgia. Her medical and family history was unremarkable. Based on vascular murmurs, abdominal aortic stenosis, and right renal artery stenosis, the diagnosis of Takayasu disease was established. Steroid pulse therapy was performed, followed by continuous treatment with prednisolone and azathioprine. At that time, her systolic blood pressure was 130 mm Hg, serum creatinine level was 1.3 mg/dL, and urinary protein level was 0.2 g/day. However, 2 months after the start of treatment, she had been experiencing lower leg edema and an elevated urinary protein level (7 g/gCr) for 2 months after treatment and admitted.

Her vital signs upon admission were normal except for blood pressure (153/106 mm Hg). Laboratory test findings were as follows: white blood cell count, 12,460/mcL; hemoglobin level, 14.7 g/dL; platelet count, 21.5 × 10^4^/mcL; albumin level, 2.4 g/dL; serum creatinine level, 1.06 mg/dL; estimated glomerular filtration rate, 58.7 mL/min/1.73m^2^; blood urea nitrogen, 36.2 mg/dL; sodium level, 136 mEq/L; potassium level, 3.4 mEq/L; chloride level, 100 mEq/L; IgG level, 287 mg/dL; IgA level, 85 mg/dL; IgM level, 74 mg/dL; CH50 level, >60.0 U/mL; C3 level, 129.5 mg/dL; C4 level, 24.1 mg/dL; renin activity, 3.2 pg/mL; and aldosterone level, 322 pg/mL. Urinary tests revealed a urinary protein-to-creatinine ratio of 16.0 g/gCr and a urinary sediment red blood cell count of 5-9/HPF.

Magnetic resonance angiography (MRA) revealed narrowing of the abdominal aorta and circumferential thickening of the vascular wall. Severe stenosis of the celiac artery, bilateral renal arteries, and inferior mesenteric artery was noted. As the root of the right renal artery could not be identified, stenosis and obstruction were suspected. In the 2 left renal arteries, stenosis was observed at the root of the artery branching from the caudal side, and the artery branching from the cranial side flowed into the upper and lower poles of the left kidney with mild stenosis at its root. The middle part of the left kidney was slightly atrophied, and mild compensatory swelling was observed in the upper and lower poles. The right kidney was 111 × 34 mm and the left kidney 124 × 42 mm. Renal scintigraphy with 99mTc revealed 10.3% uptake by the left kidney and 3.65% by the right.

A left renal open biopsy was performed, and light microscopy revealed 57 glomeruli, one with global sclerosis and 30 focal segmental glomerulosclerosis (FSGS) lesions. Immunofluorescence staining findings were unremarkable. Electron microscopy showed enlarged podocytes and endocapillary hypercellularity (Fig 1).

**Figure 1:**
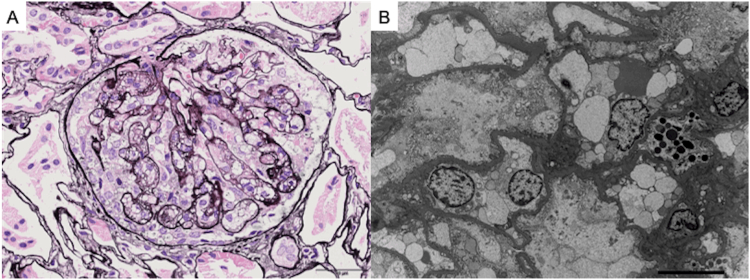
Kidney biopsy findings. (A) Light microscopy shows FSGS (cellular variant) on periodic acid methenamine-silver staining (original magnification, ×400). (B) Electron microscopy (EM) shows swelling of podocytes and increase in endothelial cells and foam cells (original magnification, ×3,000).

FSGS was considered due to hemodynamic mechanisms rather than immunologic mechanisms because urinary protein levels improved to 2.22 g/day from 8.79 g/day following antihypertensive treatment with calcium channel antagonists. Severe stenosis in the dorsal branch of the left renal artery and decreased fractional flow reserve (FFR) were noted. As renal dysfunction was observed, the decision was made to perform PTRA to improve renal function. Given that the right kidney was considerably atrophied, PTRA was conducted only for the left kidney. The procedure was performed for the ventral and dorsal branches of the left renal artery, resulting in an improvement in FFR from 0.2 to 0.95 in the dorsal branch (Fig 2). Proteinuria was also ameliorated (urinary protein: 0.7 g/gCr), and aldosterone level improved to 134 pg/mL, but renin activity was 7.2 ng/mL/hr. After initiating angiotensin receptor blocker, systolic blood pressure was between 110 and 119 mmHg, and proteinuria improved to 0.2 g/gCr; the patient achieved complete remission with improvement of blood pressure.

**Figure 2:**
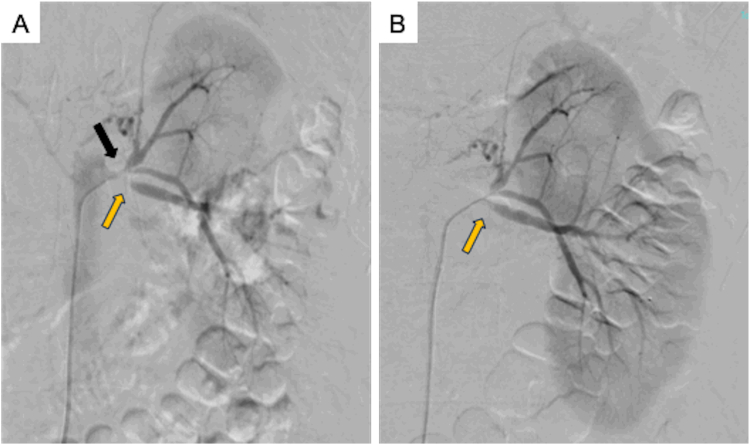
Renal artery contrast shows severe stenosis of the left renal artery at its site of origin. (A) Dorsal branch, orange arrow; ventral branch, black arrow. (B) Successful expansion of the left renal artery after PTRA.

## Discussion

Although there have been cases of nephrotic syndrome following PTRA for renal artery stenosis,^9^, ^10^, ^11^ reports on the effectiveness of PTRA for nephrotic syndrome are rare.^7^^,^^8^ In our case, the patient achieved complete remission with improvement of blood pressure after performing PTRA for renal artery stenosis with nephrotic syndrome due to Takayasu disease.

In Takayasu disease, inflammation often damages the aorta and its main branches. Typically, young women are more prone to develop the disease; female to male ratio is 8:1. Takayasu arteritis occurs more often in Asian patients, with an annual incidence rate of 1-3 per million.^12^ The occurrence of glomerulonephritis in patients with the disease is rare, and complications of lupus nephritis, IgA nephropathy, amyloidosis, membranoproliferative glomerulonephritis, and crescentic glomerulonephritis have been reported to be associated with Takayasu disease.^13^, ^14^, ^15^, ^16^, ^17^ In our study, the patient presented with FSGS, but the pathogenesis of FSGS in Takayasu disease is yet to be identified. Moreover, FSGS in Takayasu arteritis is not necessarily accompanied by renal artery stenosis and hypertension, and whether it is associated with immunologic mechanisms remains unknown.^6^

In this case, we performed an open renal biopsy in the left kidney. The tissue was taken from the lower pole, which was swollen, avoiding the central part because it was atrophic. The findings indicated excess blood flow. Collecting samples from the swollen regions on MRA is preferable to confirm the diagnosis. Identifying the central site of the pathology is crucial for diagnosis. PTRA should be performed after confirming the differences in blood flow in each kidney using a renogram and in vascular morphology using MRA, followed by biopsy and diagnosis.

The association between PTRA and nephrotic syndrome has been previously reported. Almirall et al. revealed that nephrotic syndrome was noted 3 weeks after PTRA was performed for 80% stenosis of the right renal artery in a 72-year-old male patient.^9^ Park et al^10^ reported that nephrotic syndrome was noted 4 weeks after PTRA was performed for 80% stenosis of the left renal artery in a 48-year-old male patient. Kanagasundaram et al^11^ also reported that nephrotic syndrome was noted two weeks after PTRA was performed for 95% stenosis of the right renal artery in a 65-year-old female patient. These patients possibly developed nephrotic syndrome due to renal hyperfiltration caused by stenosis removal, and all of them had chronic kidney disease with arteriosclerosis due to concomitant use of multiple antihypertensive drugs. However, in our case, nephrotic syndrome was not exacerbated with increased renal blood flow after PTRA because our patient was young with no chronic kidney disease or hypertension. Unilateral renal artery stenosis induces ischemia in the downstream kidney, resulting in the increase of renin, angiotensin II, and aldosterone. When the cascade of renin-angiotensin-aldosterone system is activated, the intraglomerular pressure of the unaffected kidney increases,^18^ which is supported by animal experiments.^19^^,^^20^ Therefore, early PTRA to normalize the cascade of renin-angiotensin-aldosterone system may be effective for renal artery stenosis accompanied by renal dysfunction.

We reported here a case of remission after PTRA for Takayasu disease complicated with renal artery stenosis presenting with nephrotic syndrome. However, the pathogenesis of PTRA-induced remission is not yet fully understood. Further research is needed to determine the efficacy of PTRA in nephrotic syndrome.

## References

[1] Delles C., Weidner S., Schobel H.P., Rupprecht H.D. (2002). Renal-artery stenosis in a patient with Takayasu’s arteritis. Nephrol Dial Transplant.

[2] Berlyne G.M., Tavill A.S., De Baker S.B. (1964). Renal artery stenosis and the nephrotic syndrome. Q J Med.

[3] Montoliu J., Botey A., Torras A., Darnell A., Revert L. (1979). Renin-induced massive proteinuria in man. Clin Nephrol.

[4] Eiser A.R., Katz S.M., Swartz C. (1982). Reversible nephrotic range proteinuria with renal artery stenosis: a clinical example of renin-associated proteinuria. Nephron.

[5] Kumar A., Shapiro A.P. (1980). Proteinuria and nephrotic syndrome induced by renin in patients with renal artery stenosis. Arch Intern Med.

[6] Tiryaki O., Buyukhatipoglu H., Onat A.M., Kervancioglu S., Cologlu S., Usalan C. (2007). Takayasu arteritis: association with focal segmental glomerulosclerosis. Clin Rheumatol.

[7] Tsuchida T., Yano H., Raita Y., Kinjo M. (2019). Nephrotic range proteinuria and metabolic alkalosis in Takayasu arteritis. BMJ Case Rep.

[8] Wakui H., Hosokawa Y., Oshikawa J. (2014). Endovascular treatment of renal artery stenosis improves contralateral renal hypertrophy with nephrotic syndrome. CEN Case Rep.

[9] Almirall J., Mendez I., Comet R., Andreu X. (2000). X: Nephrotic syndrome after renal percutaneous transluminal angioplasty. Nephrol Dial Transplant.

[10] Park H.J., Jang H.N., Cho H.S., Chang S.-H., Kim H.-J. (2019). A case report of successfully treated nephrotic syndrome after renal angioplasty. BMC Nephrol.

[11] Kanagasundaram N.S., Allan B.J., Kessel D., Newstead C.G., Worth D.P. (1998). Nephrotic syndrome after successful renal angioplasty. Nephrol Dial Transplant.

[12] Maritati F., Iannuzzella F., Pavia M.P., Pasquali S., Vaglio A. (2016). Kidney involvement in medium- and large-vessel vasculitis. J Nephrol.

[13] Sano N., Kitazawa K., Totsuka D. (2002). A case of lupus nephritis with alteration of the glomerular basement membrane associated with Takayasu’s arteritis. Clin Nephrol.

[14] Kuroda T., Ueno M., Sato H. (2006). A case of Takayasu arteritis complicated with glomerulonephropathy mimicking membranoproliferative glomerulonephritis: a case report and review of the literature. Rheumatol Int.

[15] Koda R., Yoshino A., Imanishi Y. (2014). A case of membranous glomerulonephropathy associated with Takayasu’s arteritis. Case Rep Nephrol Urol.

[16] Ateş K., Ertürk S., Diker E. (1996). Renal amyloidosis complicating Takayasu’s arteritis: a case report. Nephron.

[17] Cavatorta F., Campisi S., Trabassi E., Zollo A., Salvidio G. (1995). IgA nephropathy associated with Takayasu’s arteritis: report of a case and review of the literature. Am J Nephrol.

[18] Pandey M., Sharma R., Kanwal S.K. (2013). Hyponatremic-hypertensive syndrome: think of unilateral renal artery stenosis. Indian J Pediatr.

[19] Eisenbach G.M., Liew J.B., Boylan J.W., Manz N., Muir P. (1975). Effect of angiotensin on the filtration of protein in the rat kidney: a micropuncture study. Kidney Int.

[20] Bohrer M.P., Deen W.M., Robertson C.R., Brenner B.M. (1977). Mechanism of angiotensin II-induced proteinuria in the rat. Am J Physiol.

